# Engineered non-contestation: Deterring electoral contestation using violence in local elections

**DOI:** 10.1177/00223433251353645

**Published:** 2025-08-29

**Authors:** Noyonika Das

**Affiliations:** University of Amsterdam, the Netherlands

**Keywords:** Elections, violence, subnational democracy

## Abstract

How and why do incumbents use local elections as tools for subverting democracy and establishing party dominance? Integrating literatures on political violence and decentralization, this article argues that incumbents use violence to engineer non-contestation among opposition actors in local elections. Drawing on the strength of local networks, incumbents prioritize their own strongholds for such violence, leading candidates to withdraw and resulting in uncontested seats. I explore this argument with data on local elections from West Bengal, a state in India that has held local elections since the 1970s but where competition is spatially uneven, resulting in a significant number of uncontested seats. The theoretical expectations are tested with disaggregated data on competitiveness and uncontested seats for 3,000 local electoral units. The article finds that increased violence against the opposition in an electoral unit leads to seats going uncontested in that unit. I complement these findings with 60 qualitative interviews from political elites and non-elites, which indicate that violence is an important means through which incumbents engineer non-contestation. These findings have important implications for research on political violence and subnational authoritarianism, particularly in understanding the emergence and persistence of subnational authoritarianism in decentralized countries.

## Introduction

Competitive elections are an essential feature of democracy, empowering citizens to hold leaders accountable and shape governance. However, uncontested electoral seats – especially in local elections – undermine these principles, creating a democratic deficit. This issue worsens when violence is used strategically to deter opposition candidates, securing uncontested victories for incumbents. Such tactics deny voters meaningful choice and weaken the integrity of local democratic institutions, often seen as foundations of participatory governance (Mahmood, 2024). Urgency to understand this issue is heightened by the rising incidence of electoral violence and uncontested seats in democracies across the Global South (Bartels et al., 2023; Cruz, 2023).^1^ These trends are puzzling as opposition parties often contest elections despite significant constraints, using the opportunity to criticize incumbents even when they stand little chance of winning (Daxecker and Rauschenbach, 2023). Meanwhile, incumbents often permit opposition participation to showcase the appearance of free and fair elections, thus gaining legitimacy (Bertrand, 2021). Therefore, despite clear benefits to both the incumbent and the opposition, the prevalence of uncontested seats raises numerous questions with implications for the health and future of local democracy.

This article advances our understanding of where and how non-contestation in local elections occurs, offering a novel perspective that it can be actively and coercively engineered. I argue that non-contestation is not merely a reflection of opposition candidates’ choices or limited political participation but also an outcome of provincial incumbents’ electoral strategies, often involving extreme acts of violence such as killings, kidnappings, or threats, which force opposition candidates to withdraw (*ACLED*, 2024). Specifically, I contend that variations in incumbent strength shape the likelihood of non-contestation, with uncontested seats more likely in areas where incumbents have strong networks. Additionally, I highlight violence as an underappreciated driver of non-contestation, paradoxically occurring in areas already under incumbent control. While prior research on ‘silent elections’ emphasizes incumbency advantage, where challengers avoid contests to conserve resources, this article extends the inquiry by examining the role of violence (Caramani, 2003; Harbers, 2014). It seeks to answer why winning by a large margin is not sufficient for incumbents and how violence is used to engineer non-contestation, indicating that non-contestation can be purposefully engineered to establish hegemony rather than being an unfortunate outcome of the opposition’s lack of organization or resources.

This article tests this argument using the case of West Bengal, a state of over 100 million people in India. West Bengal provides a compelling case because it holds regular local elections often marred by illicit strategies, particularly violence. Moreover, competition in the state has remained spatially uneven, marked by a high prevalence of uncontested seats – a strategy consistently utilized by incumbents over the years (Nath, 2019). Using a mixed-method approach with a novel dataset, the article examines how provincial incumbents leverage their partisan apparatus to orchestrate violence and deter opposition candidates from contesting in local elections. The analysis incorporates original election results data from over 3000 local electoral units, revealing that areas dominated by incumbents are more likely to experience non-contestation. Additionally, it draws on new disaggregated and georeferenced event data on violence during the 2018 local elections, enabling an analysis of the spatial variation and targets of violence. The quantitative findings are further supported by 60 interviews with political actors and ordinary voters, which corroborate the results and provide nuanced insights into how violence functions as the means to achieve non-contestation.

This article makes two key contributions to our understanding of local politics and election violence. First, it reconceptualizes uncontested seats as a deliberately engineered outcome of the incumbent’s electoral strategy. Existing research suggests that incumbents’ access to resources gives them a competitive advantage, leading challengers to withdraw from races they cannot win (Konisky and Ueda, 2011). Alternatively, non-contestation is sometimes framed as an issue of representation, such as in cases of minority candidate emergence (Tolley, 2019). These perspectives, however, neglect how incumbents can exploit their position to engineer non-contestation among opponents, further solidifying their power. This reconceptualization has significant theoretical implications, as it highlights that non-contestation does not equate to a total lack of competition. Instead, it points to a context where opposition candidates are willing to contest but are forcibly excluded. This purposeful exclusion of competitors undermines the democratic process of elections, forcing voters to accept predetermined outcomes and reducing the political uncertainty inherent in elections(Schedler, 2013).

Second, by integrating literatures on local politics and political violence to develop the argument, the article explains how engineered non-contestation is achieved violently. While political violence has long been used as a tool for electoral demobilization, recent years have seen an increase in violence targeting political actors, particularly at the local level (Kumar, 2020). For instance, India experienced a 44% increase in violence against local officials in 2023 compared to 2022, with significant spikes in violence during elections in states such as West Bengal, Manipur and Uttar Pradesh. These trends highlight a growing correlation between elections and targeted violence (Harish and Toha, 2019). Despite the acknowledged importance of local elections in shaping grassroots power dynamics, research on local politics often overlooks the role of violence, particularly in rural settings, with much of the existing work focusing on urban contexts (Wilkinson, 2004). Using both qualitative and quantitative sources of data the article shows how violence is engineered to produce non-contestation and provides more nuance to understanding the increased levels of violence in local politics.

### What do we know about uncontested seats?

An uncontested seat can be defined as an electoral seat where only one candidate runs, leaving voters with no alternative option (Kouba and Lysek, 2023). Consequently, voters are unable to express their preferences through voting as the sole contender wins the election uncontested. Thus, elections with uncontested seats pose a greater concern compared to elections with uncompetitive seats. In uncontested elections, voters are deprived of any influence on the outcome, as the candidate automatically secures victory without any votes being cast. In contrast, uncompetitive elections, while lacking meaningful competition, still feature few challengers, theoretically allowing voters to make a choice (Konisky and Ueda, 2011; Schedler, 2002). Even in rigged elections, citizens can signal disapproval by abstaining or spoiling ballots, options unavailable in uncontested elections (Frantz, 2018; Power and Roberts, 1995).

Prevailing explanations for uncontested seats often suggest that opposition actors opt to withdraw from electoral races when faced with significantly stronger competitors (Carey et al., 2000; Lay et al., 2017; Squire 2000). While, theoretically, any party or competitor could deter challengers from contesting, incumbents typically possess the greatest capacity to exert such influence. The occurrence of uncontested seats is frequently attributed to the financial and political resources enjoyed by incumbents. Research on uncontested seats within subnational state legislatures has shown that legislatures provide incumbents with ample means to solidify their positions and increase their electoral advantage (Konisky and Ueda, 2011).

Other explanations highlight disparities in political representation, particularly for marginalized groups. In contexts where incumbents often belong to dominant groups, minority challengers are less likely to emerge due to structural and systemic disadvantages (Tolley, 2019). In India, quotas for marginalized groups shape who can run for office. Marginalized groups, often constrained by economic and social barriers, are less likely to produce strong candidates. As a result, the pool of contenders for reserved seats is often limited, with most strong candidates aligning with incumbents, further reinforcing their advantage (Auerbach and Ziegfeld, 2020). While these explanations provide insight into non-contestation, they overlook a critical cause: non-contestation as a deliberate incumbent strategy, often enforced through violence.

A second strand of literature, moving away from incumbency advantage, emphasizes the influence of political parties as organizational entities on levels of contestation. This body of work traces the transition from an elite-dominated mode of politics to one where political parties dominate. For example, a restricted electoral franchise and dominant local notables partly explain the large number of uncontested seats in electoral contests in the UK (Foster, 1977; Sharman, 2003). Similarly, a study of British parliamentary elections found that incumbents with ties to the nobility were more likely to remain unchallenged (Coats and Dalton, 1992). Other cross-national studies argue that changes in levels of non-contestation are congruent with changes in party organization (Rallings et al., 2005). The emergence of modern parties focusing on ‘systemwide’ campaigning rather than local community politics has been linked to rising levels of contestation (Caramani, 2003; Sharman, 2003). However, this cannot explain the recent worrying rise in uncontested elections around the world.

Recent research highlights the instrumentalization of political parties for violence, where parties are mobilized to demobilize rivals through coercive strategies (Klaus and Turnbull, 2025; Malik, 2024; Siddiqui, 2022). This is particularly relevant in understanding the absence of contenders in local elections. Theories of political violence provide useful insights into the ‘exclusion of competitors’ from the electoral field, with scholarship highlighting how opponents are demobilized through various illicit methods to tilt the playing field in favor of the incumbent (Birch et al., 2020; Harbers et al., 2022; Schedler, 2002). Research on incumbent-sponsored election violence finds that a majority of the violence is directed at making opposition mobilization more difficult (Gandhi and Lust-Okar, 2009; Taylor et al., 2017). In such contexts,where the incumbent is seeking re-election most of the violence occurs in the pre-electoral period (Bhasin and Gandhi, 2013; Hafner-Burton et al., 2018). While most of these studies focus on incumbent-sponsored violence at the national level, similar empirical findings have been made at the subnational level (Berenschot, 2011; Harish and Toha, 2019; Turnbull, 2020).

Incumbents at both the national and subnational level have been implicated in the targeting of challengers and their supporters, which constitutes a form of ‘electoral cleansing’ (Bratton and Masunungure, 2008). This subcategory of elite-upon-elite violence has primarily focused on lethal events and assassinations (Perliger, 2017). The targeted assassinations of candidates appears to occur primarily in the pre-electoral phase to skew the electoral outcome by eliminating threats to political power (Onapajo, 2014). This lethal violence against opposition elites has certain spillover effects, where violence against the opposition signals to the electorate the inability to protect itself. Incumbent-sponsored violence diminishes confidence in the opposition as a viable alternative by making them appear weak (Edwards and Pierson, 2022).

While the research on incumbent-sponsored violence is abundant, it is still unclear whether and how political violence and uncontested seats are geographically related and why that is the case. Studies on election violence at the subnational level indicate that violence is more likely in uncompetitive units rather than competitive ones, as it is used to signal political dominance (Toha, 2017; Wahman and Goldring, 2020). However, it is uncertain who controls these uncompetitive units – the opposition or the incumbent. Some studies find evidence of violence in incumbent strongholds, while others find evidence in favor of opposition strongholds (Albarracín et al., 2022; Choi and Raleigh, 2021; Daxecker and Rauschenbach, 2023; Wahman, 2023).

Existing explanations of electoral violence often focus on how opponents are weakened during elections, overlooking how they can be entirely incapacitated from contesting. Some findings emerge from studies on subnational undemocratic regimes, which reveal that such regimes that attempt to demobilize the opposition often lack commitment to principles of good governance and political accountability (Gibson, 2005; Giraudy, 2015). This scholarship demonstrates that provincial incumbents employ strategies of ‘boundary control’ to insulate their local control, yet these insights have not been fully integrated into our understanding of why and how non-contestation occurs (Behrend, 2011). There is limited systematic evidence illustrating the violent processes underlying the production of uncontested elections, which serves to consolidate the position of subnational incumbents.

### A theory of engineered non-contestation

There is limited understanding of the use of violent strategies to deter challengers from contesting in local elections and how this contributes to consolidating local electoral control. This article presents a theory to explain why and how provincial incumbents resort to such measures to engineer non-contestation and secure their victory, with violence being a crucial element in this process. The focus is solely on incumbent calculations and not on the opposition’s response. Specifically, this theory explains why winning by a large margin is not sufficient for the incumbent and how violence drives non-contestation. Provincial incumbents use violence to engineer non-contestation during local elections, consolidating their control, with the violence paradoxically being most prevalent in their strongholds. These strategies create arenas of illiberal politics, where the incumbent party asserts dominance over local affairs while impeding opposition mobilization efforts. By doing so, incumbents strengthen stronghold boundaries, reducing the likelihood of opposition advances supported by national partisan allies (Gibson, 2005). The degree of central government intervention in subnational politics can vary across countries, necessitating provincial incumbents to devise strategies to neutralize such threats. In India, for instance, the imposition of President’s Rule allows the central government to assume control over a province, posing a vertical threat to provincial autonomy (Harbers et al., 2019).^2^ Within this context, violence by provincial incumbents can also be interpreted as a defensive strategy to weaken rivals within, and consolidating their power to resist central interference.

In this article, the provincial incumbent refers to a political party in power at a subnational level, operating below the national level. With decentralization reforms, many countries now have provincial incumbents wielding significant fiscal, administrative and political authority over their provinces. These second-tier provincial incumbents may resort to various undemocratic actions to prolong their regime continuity, including preventing the opposition from attaining any elected office (Giraudy, 2015). In India, provincial incumbents have frequently been linked to violence, particularly when it yields electoral advantage (Daxecker et al., 2024; Wilkinson, 2004). Subnational democracy in India varies, with many provincial parties asserting hegemonic control by employing violence and limiting voters’ ability to choose viable opposition alternatives (Harbers et al., 2019).

Local elections present viable opportunities to strengthen or weaken degrees of political control through effective campaign strategies. Although distinct from rebel groups, provincial incumbent parties similarly use violence to assert territorial dominance for both symbolic and strategic purposes (Kalyvas, 2006). It is essential to note that political power in rural or peripheral areas of developing democracies is often intertwined with illicit sources of income. A loss in local elections to the opposition not only represents a formal electoral defeat but also threatens access to these revenue sources and the patronage networks that sustain political dominance (Albarracín et al., 2022). These revenue streams play a pivotal role in rewarding loyal supporters, financing election campaigns and sustaining violence capabilities. Provincial incumbents frequently rely on violent extortive methods to retain control over illicit resources as they compete with partisan rivals (Martin and Michelutti, 2017; Vaishnav, 2017). Local elections, therefore, become a battleground where opposition parties can establish grassroots political control, posing a direct threat to the incumbent’s dominance. It poses a twofold threat: it undermines the ruling party’s control over local resources, and disrupts its future electoral prospects. The heightened stakes and being the one with the most to lose explain why non-contestation is engineered using violence to reduce electoral uncertainty.

The violent engineering of non-contestation fulfills the dual objectives of weakening the opposition and establishing a hegemonic presence. Mere manipulation through vote-buying or other clientelist strategies is often insufficient, especially during local elections that, while skewed in the incumbent’s favor, pose a real threat to the existing status quo. Using violence to win elections and dominate the political landscape is crucial for provincial incumbents who must navigate both local and national politics. National elites have significant resources to support the electoral campaigns of their provincial affiliates or can deploy state forces to suppress provincial rivals. Building a strong local foundation gives provincial elites greater leverage and helps keep the central government out of local politics (Gibson, 2005). While constitutional safeguards limit the provincial incumbent’s ability to directly violate democratic institutions extensively, it can utilize its partisan apparatus to circumvent formal rules. The costs of outright banning opposition activity are high, leading the provincial incumbent to potentially use non-state actors, like party workers, to carry out violence on its behalf (Levitsky and Way, 2010; Turnbull, 2020).^3^

Through violence, the incumbent aims to dismantle the local infrastructure of rival parties, refusing to tolerate any form of rival party mobilization. Therefore, this article theorizes the logic behind targeting the opposition. The persistent use of violence against any rival presence makes it exceedingly difficult to sustain opposition efforts. Violence is often preferred over nonviolent strategies as it provides a swift and decisive way to disrupt opposition activities, achieving results faster than legal or administrative barriers. Buying off competitors is resource-intensive, and involves risks as opposition actors can reject offers. In contexts with weak judicial or bureaucratic oversight, violence becomes a more accessible tool for incumbents. Opposition leaders and party workers, being a smaller group compared to ordinary voters, are easier to identify and target with violence. Additionally, opposition actors are more visible during electoral campaigns. Targeting them with violence acts as a form of ‘electoral cleansing’, which eliminates electoral threats and cripples opposition collective action (Bratton and Masunungure, 2008).

By limiting the targets of violence, the incumbent maintains its credibility. The use of violence against opponents may not elicit a backlash from the electorate if they are not directly affected. Violence against the opposition in the pre-electoral period is less likely to make voters doubt the fairness of elections, as they do not experience the full effects of violence (Bhasin and Gandhi, 2013).^4^ Thus, this article hypothesizes that opposition leaders and workers will be the primary targets of violence, focusing on the subcategory of elite-upon-elite violence (Kongkirati, 2016; Schedler, 2002).

*Hypothesis 1*: Increased electoral violence against the opposition in an electoral unit leads to a higher number of uncontested seats in that unit.

However, resource constraints may prevent this strategy from being implemented everywhere. Local political geography will dictate how election violence will play out (Müller-Crepon, 2022; Wahman, 2023). The use of violence in areas already controlled by the incumbent party may seem counterintuitive, but it is deemed necessary for ‘boundary control’ and safeguarding the territories they control (Behrend, 2011; Gibson, 2005). This article anticipates that violence for establishing a hegemonic presence will predominantly occur within incumbent strongholds, a pattern also documented in many contexts like Colombia (Uribe, 2025). In strongholds, the incumbent can closely monitor violence, enforce compliance and effectively punish non-compliance. As incumbents, they leverage dense partisan networks and local dominance, often reinforced by control over police and judicial entities, enabling them to orchestrate violence with minimal repercussions (Berenschot, 2020; Daxecker et al., 2024).

Moreover, rival parties in such strongholds are typically poorly organized. The incumbent can effectively use violence to coerce opposition candidates into withdrawing their candidatures. In these areas, where the opposition lacks significant support and security resources, they are unable to counter threats or mobilize voters who fear openly opposing the incumbent. Additionally, violence in strongholds faces less resistance from voters, as a majority who support the incumbent perceive violence by co-partisans as minor infractions (Daxecker and Fjelde, 2022). More importantly, violence in this context goes beyond securing an election win. Ultimately, such violence deepens partisan control and entrenches political dominance, consolidating the incumbent’s hold on power (LeBas, 2006; Nath and Ray, 2022). With no opposition, fewer resources are needed for strategies like vote-buying, freeing finances to target competitive areas. Thus, a second hypothesis is

*Hypothesis 2*: Violence against the opposition increases uncontested seats more in electoral units where the incumbent is stronger than in units it is not.

The argument outlined above has certain scope conditions. The theory and the empirics of this article are limited to the incumbent’s capacity and incentives for bringing about non-contestation. What is more, the article looks at the strategies used by a provincial-level incumbent, having a distinct political aim, in a decentralized electoral system. The political aim is the desire to consolidate control within its province. While opposition actors may have incentives for violence, incumbents face higher stakes, as losing local control threatens their broader political future in subnational elections. Therefore, the article excludes opposition calculations from the argument.

The theory developed in this study, based on India, has important implications for generalizability. This article develops a general theory of interaction between the provincial incumbent and local politics. It is not confined to understanding violence in contexts with a three-tiered system of government, like India. At its core, the theory is a two-level theory, as insights from this article can also be used to understand violent dynamics in two-tiered systems of governance where the subnational incumbent attempts to consolidate control using violence. It is particularly applicable to contexts where decentralization has endowed subnational units with substantial fiscal and political autonomy.

The implications of this framework extend to contexts where decentralization fosters entrenched systems of clientelism and patronage, as political power provides access to economic opportunities and legal immunity. Losing power in such systems risks not only financial losses but also exposure to legal repercussions (Birch, 2021). These dynamics are particularly pronounced in countries like Zimbabwe and Indonesia, where violence against candidates – especially during the nomination process – has been a recurring phenomenon (Seeberg et al., 2020; Toha, 2017).^5^ Moreover, the compromised independence of electoral institutions under decentralized systems, as seen in India and Ethiopia, further facilitates incumbent-sponsored violence (Fjelde and Höglund, 2016; Mahmood, 2020). Additionally, in emerging democracies, the age of democracy and constitutional reforms can introduce new drivers of violence, amplifying the risks associated with weak institutional checks (Malik, 2018; Von Borzyskowski et al., 2022). While not universally applicable, the findings offer insights into low-accountability contexts with competitive local elections that facilitate opposition exclusion.

## Empirical context

### The local governance system

The eastern province of West Bengal, bordering Bangladesh, Nepal and Bhutan, is India’s fourth most populous state and a pioneer in decentralization, devolving power to local levels 14 years before the 73rd Constitutional Amendment in 1992.^6^

The system of rural local governance in West Bengal is three tiered. The lowest and smallest unit in the system is the local village council also known as the *Gram Panchayat* (GP). The GP is divided into wards and voters elect a candidate from within their wards to a seat within the GP.^7^ Therefore, the GP consists of an elected member from each ward. Unlike most other Indian states, these elected councillors have party affiliations.^8^

The Left Front government introduced this three-tiered system partly in response to state repression during the Emergency (1975–1977) and partly to consolidate rural support through land reforms (Kohli, 1991).^9^ While local offices served as crucial channels for implementing rural welfare schemes, the primary motive behind decentralization was to maintain the party’s control over the rural electorate. Electoral integrity was often undermined through incentives and violence, including engineered non-contestation (Ghosh and Sahoo, 2022). Growing voter dissatisfaction with these coercive practices facilitated the rise of the All India Trinamool Congress (AITC).

Like its predecessors, the AITC used local councils to implement welfare schemes alongside coercive tactics. Distributing direct cash benefits, food grains and other perks, the AITC secured rural support and a historic mandate in the 2016 elections. However, these strategies were marred by violence, corruption and extortion. The party co-opted local strongmen, leveraging their influence to maintain control through coercion and extractive practices (Bhattacharyya, 2023).

In addition to coercive patronage, the AITC engineered non-contestation using violence. Uncontested seats have been a recurring feature in rural elections. For instance, 11% of seats were uncontested in 2003, falling to 5% in 2008, but rising again to 10.6% in 2013; and then 34% in 2018 by the AITC (Bhattacharyya and Rana, 2013; Times of India, 2018e). The 2018 elections sparked widespread concern as well as inquiry by the media and judiciary (The Indian Express, 2018). The political landscape was rife with accusations of violence by opposition party members and several complaints were filed against the incumbent. Opposition parties filed requests to submit their nomination papers online as they claimed that they were unable to do so in person (Times of India, 2018a). Candidates and their supporters were assaulted en route to Block Development Officer headquarters^10^ (Times of India, 2018d).

## Research design

### Case selection

While this article focuses on West Bengal, similar patterns of violence and manipulation during local elections are visible across India. Although extreme violence may be less frequently reported elsewhere, local elections across India are not immune to fraud. Local elections, administered by more vulnerable State Election Commissions, are prone to provincial incumbent interference, including postponements or cancellations (Kumar, 2020; Martin and Picherit, 2020). Historically, local elections in states like Uttar Pradesh have also been marked by violence. Alongside overt violence, a significant number of uncontested seats have been a recurring feature in other states like Haryana and Uttar Pradesh. Violence often underpins these outcomes, as seen in Punjab, where Congress workers avoided campaigning due to threats from the incumbent (Martin, 2020; Thomas, 2016).

Given these undemocratic trends in local politics in India, West Bengal serves as a suitable case for testing the theory for three reasons. First, West Bengal has a highly decentralized electoral system. Rural local elections have been a regular feature of political life ever since their introduction in 1978 by the Left Front government. The allocation of a substantial amount of funds and administrative power to implement developmental schemes has resulted in local rural electoral units becoming extremely vital and competitive (Kumar and Ghosh, 1996). Control over such institutions allows effective party institutionalization at the local level but also the opportunity to consolidate existing patronage networks with access to state and national resources. Local elections involve elections to either municipalities in urban areas or village councils in rural areas.^11^

Secondly, even though West Bengal has held local elections since the 1970s, competition remains spatially uneven, resulting in a significant number of uncontested seats. Non-contestation has been a persistent feature of local politics in West Bengal, raising concerns about its role in reinforcing the incumbent’s hegemony and sparking debates over its impact on democratic governance (Banerjee, 2011). The high number of uncontested victories in 2018 drew attention, particularly due to accompanying allegations of electoral manipulation by the incumbent.

Finally, local politics in West Bengal appears to be dominated primarily by the incumbent. The incumbent has been implicated in the use of coercive strategies with the aim of skewing the electoral playing field. There have been reports of the incumbent routinely targeting and harassing its opponents (Daxecker and Fjelde, 2022; Nath, 2019). While the incumbent is motivated to use extralegal measures to secure a victory, the elections in the state are free and fair to some degree. The democratic institutions in place cannot wholly be usurped by the provincial incumbent to guarantee a victory. The intervention of the judiciary and the involvement of security forces from outside the state safeguard the rights of opponents to some extent. The existence of the secret ballot in all elections safeguards the rights of voters (Times of India, 2018c).

## Data and methods

### Quantitative data

Given the paucity of fine-grained data on violence during local elections, a multimethod approach is employed to analyze the case of engineered non-contestation. To first understand the patterns of non-contestation, the study relies on a dataset of local election results of all local rural councils in West Bengal from 2013 and 2018. The unit of analysis in this article is local rural councils: GPs. The dependent variable in this article is the proportion of *Uncontested seats* within a local council (Shenoy and Zimmermann, 2021). A seat is regarded as uncontested if there is only one candidate contesting and no votes are cast. In such cases, no election is held, and the sole candidate wins automatically. The variable *Uncontested seats* is measured as a total number of uncontested seats within a local council divided by the total number of seats in the local council. On average, 0.33 seats are uncontested within a local council.^12^ It is important to note that for the 218 local elections, all uncontested seats had the incumbent as the sole contender.

To identify whether violence was indeed driving non-contestation, I hand coded more than 900 newspaper articles. Given that violence, especially during local elections will be underreported, I relied on province and district level Bengali-language newspapers. I also relied on national and English language newspapers. These were accessed via Nexis Uni, where reports were identified using key terms such as ‘clash’, ‘riot’, ‘violence’ and so forth. Bengali-language newspapers were accessed through a local NGO archive, which did not support keyword searches (Daxecker, 2024).

The dataset comprises incidents of electoral violence that took place 6 months prior to the poll and 3 months after it. A contentious event involves at least two actors on opposite sides. Incidents of election violence coded include instances of injury and assault of opposition party and independent candidates trying to contest in elections, to setting ballot boxes on fire on election day by the incumbent party or its supporters.^13^ Some incidents of violence escalated into partisan clashes as incumbents and opposition actors clashed to be able to file their nomination papers. Actors are coded as party actors if the article mentions individuals as ‘activists, members or workers’ of a party. The partisan affiliation, if mentioned, is also noted. For the purposes of this article, the focus is only on violence that occurred in the pre-electoral period, that is, until the period for filing nomination papers and withdrawal was over. Therefore, there were a total of 165 violent events in the pre-electoral period. On average, 0.27 local councils had at least one violent event within a distance of 3 km. The results of other distance specifications are reported in Online Appendix A.

Despite recent criticism of newspaper-based datasets in the study of election violence, newspapers are relied upon in this study because they provide the best available data on election violence, especially for local elections, which are not often widely reported or analyzed (Von Borzyskowski and Wahman, 2021).^14^ To mitigate the effects of underreporting, multiple newspaper archives were consulted, as they are considered the best source. Government data can sometimes downplay the incidence of violence sponsored by the incumbent or may not provide micro-level information.

While this dataset provides valuable insights into electoral violence, key challenges to inference remain. Underreporting, especially in rural areas, may obscure local-level intimidation that is not consistently documented. Although reliance on multiple newspaper archives, including Bengali-language outlets, mitigates this to some extent, biases inherent in media reporting persist. Selective coverage and editorial constraints may overrepresent newsworthy incidents, underestimating less overt forms of violence. Additionally, incumbent pressures often prevent the partisan identities of actors from being reported, even when events are documented, obscuring patterns of incumbent-sponsored violence. Large-scale incidents may also be omitted from coverage due to fears of inciting further unrest in volatile areas. While the dataset provides a strong foundation, these limitations highlight the importance of cautious interpretation and acknowledgment of potential biases and missing data.

**Figure 1.**
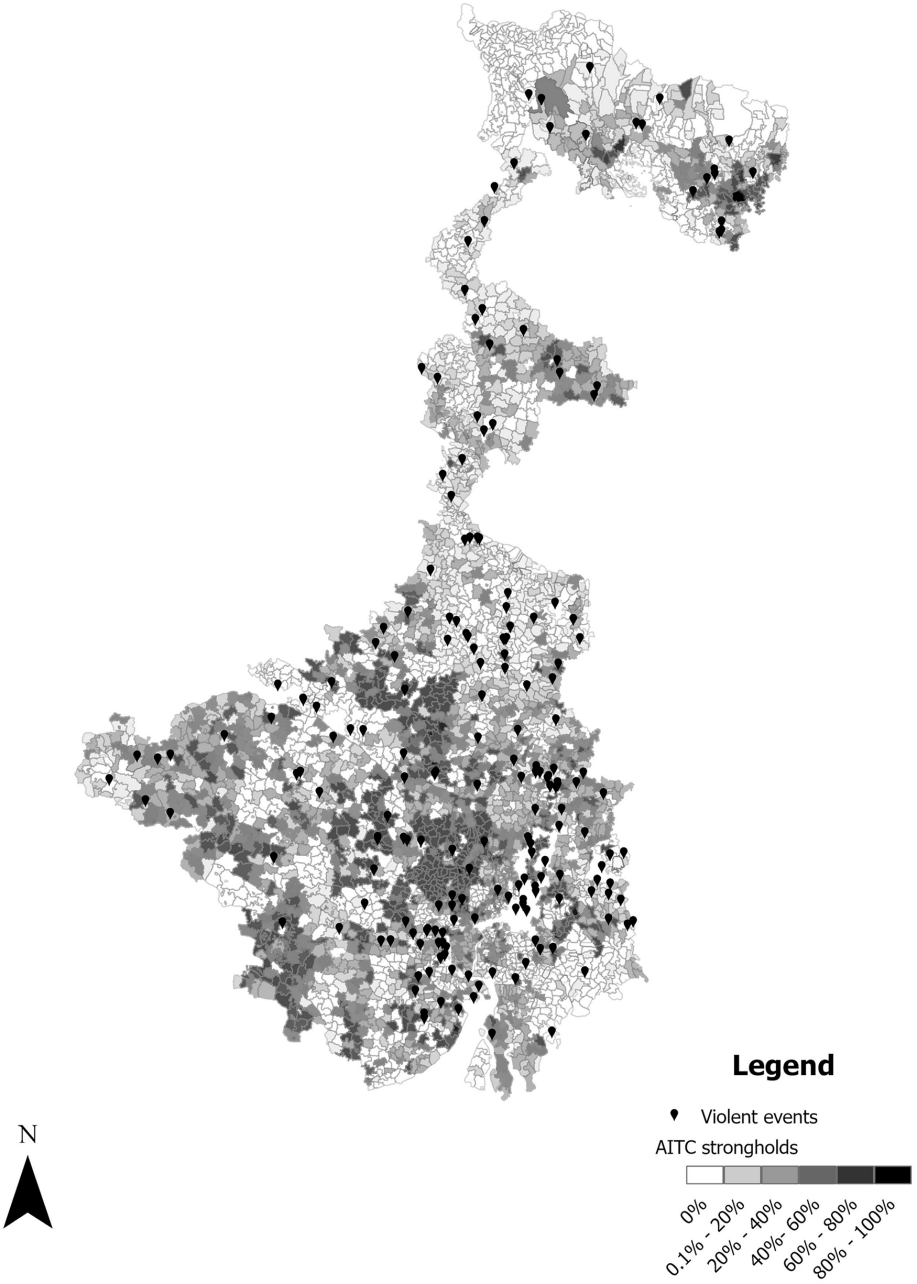
Spatial distribution of AITC strongholds and violence across West Bengal.

Figure 1 provides a visual representation of the relationship between uncontested seats and violent events. Leading up to the final day for filing nomination papers on 29 April, there was a total of six deaths. Four of these six deaths were clearly identifiable as partisan actors.

To determine whether non-contestation primarily occurs in areas controlled by the incumbent, a measure of incumbent strength is constructed using the same electoral dataset. The incumbent is assumed to evaluate prior election results to identify its strongholds, gaining insight into areas that may require violence to consolidate control. The electoral results of the 2013 local elections are used to identify incumbent strongholds and create a variable *Seats won by AITC* (2013), measured by the number of seats won in the local council election divided by the total number of seats contested in the local council.^15^ On average, the incumbent AITC won 0.42 seats through elections in a local council.

The article controls for several variables that could affect the competitiveness of a local council and/or violence. Some control variables, such as income and population, were obtained from the SEDAC dataset, while the results of the 2013 local elections were also incorporated (Meiyappan et al., 2018).

From the election results of the 2013 local elections, a variable was extracted: the proportion of *Seats reserved for minorities* (women and other backward groups). The proportion of *Seats reserved for minorities* is controlled for, as research has shown that affirmative action can influence competition and electoral outcomes (Das et al., 2023). Seats reserved for minorities can be easily manipulated by political parties, as disadvantaged groups often lack the resources or capacity to resist.

Secondly, including variables such as the *Average income* of the local council and *Total population* as controls in the analysis is a prudent approach to account for potential confounding factors. Economic development may indeed influence the likelihood of violence occurrence, as areas with higher income levels may attract more attention from political actors seeking to control resources (Jensen and Wantchekon, 2004). Similarly, densely populated areas may present more opportunities for violence due to the presence of more potential targets and better infrastructure for mobilization.

Thirdly, a measure of *Uncontested seats from 2013* is created. This variable is included because areas with a prior history of non-contestation may be more sensitive to electoral malpractices in future elections. By controlling for past non-contestation, the analysis aims to account for potential endogeneity.

Finally, I use the electoral results of the 2016 provincial assembly elections to identify the proportion of *Elected incumbent legislators in the provincial parliament*. This measure is included to control for partisan strength in districts where the incumbent performed well, as these areas may have greater capacity for violence or more campaign resources in local elections (Myneta, 2016).^16^

## Empirical analyses

### Quantitative results

Table 1 reports the baseline ordinary least squares models. The dependent variable is the *Share of uncontested seats in each GP in 2018*. The only difference across the three columns is the violence radius (3 km, 5 km, 10 km). Each model incorporates several control variables and district fixed effects, with clustered standard errors by local council to ensure robustness. It is important to emphasize that results represent associations rather than causal effects.^17^

**Table 1.** OLS regression of uncontested seats and electoral violence.

	(1)	(2)	(3)
Electoral violence (3 km)	0.020^*^ (0.009)		
Electoral violence (5 km)		0.020^**^ (0.006)	
Electoral violence (10 km)			0.015^***^ (0.004)
Controls	Yes	Yes	Yes
Constant	9.243^***^ (0.985)	9.137^***^ (0.984)	9.034^***^ (0.978)
*N*	2,330	2,330	2,330
*R*-squared	0.425	0.427	0.429

Clustered standard errors by local council in parentheses. Fixed effects by district.

† *p* < 0.10; ^*^p < 0.05; ^**^p < 0.01; ^***^p < 0.001.

Consistent with H1, electoral violence is positively and significantly associated with the share of uncontested seats at every distance. This indicates that a violence event within a 3 km and 5 km radius is associated with a 2% increase in the number of uncontested seats. Additionally, the coefficient for electoral violence within a 10 km radius is 0.015 and significant at the 1% level, implying that a violent event within this distance is associated with a 1.5% increase in uncontested seats. The smaller coefficient for violence at a 10 km radius compared to 3 km and 5 km suggests that the immediate and direct impact of electoral violence on uncontested seats diminishes with distance.

**Figure 2.**
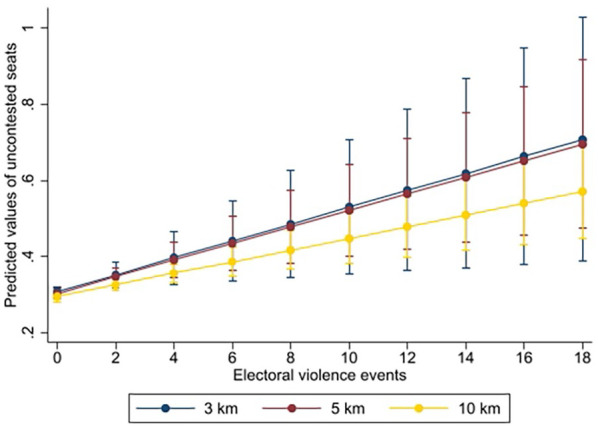
Predicted values of uncontested seats based on electoral violence at different distances.

Figure 2 visualizes these baseline results, plotting the model-predicted share of uncontested seats as violence accumulates at each radius. The steeper slope at 3 km mirrors the larger coefficient in Table 1, while the flatter 10 km line illustrates the diminishing impact with distance.

Hypothesis 2 expects that violence against the opposition increases uncontested seats more in electoral units where the incumbent is stronger. Table 2 examines H2 by interacting violence with incumbent strength (2013 AITC seat-share, mean-centered). The variables for *Electoral violence* and incumbent strength (*Seats won by AITC in 2013*) were centered. This is because the interacting variable takes on a wide range of values. The main effect of a violence event does not represent its average effect across the data, but only when *Seats won by AITC in 2013* is zero.^18^

**Table 2.** Impact of electoral violence and AITC strength on uncontested seats: Interaction effects.

	(1)	(2)	(3)
Seats won by AITC (2013) (centered)	0.069^**^ (0.025)	0.068^**^ (0.025)	0.069^**^ (0.025)
Electoral violence (3 km) (centered)	0.019^*^ (0.009)		
Electoral violence (3 km) × seats won by AITC (2013)	−0.004 (0.026)		
Electoral violence (5 km) (centered)		0.020^**^ (0.006)	
Electoral violence (5 km) × seats won by AITC (2013)		0.009 (0.017)	
Electoral violence (10 km) (centered)			0.015^***^ (0.004)
Electoral violence (10 km) × seats won by AITC (2013)			0.019 (0.010)
Controls	Yes	Yes	Yes
Constant	9.273^***^ (0.982)	9.186^***^ (0.980)	9.198^***^ (0.972)
*N*	2,330	2,330	2,330
*R*-squared	0.425	0.427	0.430

Clustered standard errors by local council in parentheses. Fixed effects by district.

† *p* < 0.10; ^*^p < 0.05; ^**^p < 0.01; ^***^p < 0.001.

The results in Table 2 suggest that a stronger incumbent presence is consistently associated with more uncontested seats. Electoral violence within 3 km, 5 km, and 10 km radii is associated with an increase in uncontested seats. However, the interaction between electoral violence and incumbent strength is significant only at 10 km. These results do not show consistent and robust findings for Hypothesis 2. These mixed findings for Hypothesis 2 may stem from the distribution and variability of violent incidents at different distances. Substantively, this suggests that opposition candidates in incumbent strongholds likely anticipate deterrence and are risk-averse due to low winning chances. However, widespread violence within a 10 km radius amplifies fear, making it harder for opposition candidates to dismiss the threat. Interestingly, the results show that districts where the incumbent party performed strongly in the 2016 provincial elections, featured fewer uncontested seats in the 2018 election.^19^

## Data and methods

### Qualitative data

In addition to relying on this novel dataset and quantitative analyses, I also undertook fieldwork in West Bengal for 6 months from 2021 to 2023. Its purpose was to contextualize and verify conclusions drawn from quantitative analyses. During the fieldwork, I conducted 60 interviews in cities, towns and 30 local councils. By selecting the most typical cases I was able to detect how violence unfolds. Typical cases serve an exploratory role, and in this case, they helped assess the plausibility of my argument. My goal was to understand the mechanisms of incumbent-sponsored violence. However, researchers often combine multiple case-selection strategies. While my cases were extreme – in that I selected cases with high levels of non-contestation – they were also typical in other respects, such as electoral competition, the size of local councils and the rural dynamics of the area (Gerring, 2006). This approach allowed me to better understand the dynamics of violence, as the puzzle of interest – how violence is produced – lay within these typical cases.

These interviews with academics, journalists, government officers and political leaders, categorized as elite interviews, were primarily conducted in urban areas where political elites are typically concentrated. Political leaders and government officers were asked about decisionmaking processes, strategies for electoral campaigns, and their views on the role of violence in shaping election outcomes. Academics and journalists were questioned about the broader political landscape, including power structures, historical trends, and their observations on the dynamics of local elections. These discussions provided valuable insights into both the organizational and structural aspects of West Bengal’s political environment.

With a deeper understanding of the context, I conducted more in-depth fieldwork in incumbent strongholds that experienced non-contestation in 2018. Interviews conducted in local councils with ordinary voters, local party workers and even local strongmen allowed me to develop a deeper understanding of local politics. These conversations with local party workers allowed me to gain insight into how violence is orchestrated and the logistics associated with planning such violence. Local government officers provided insight into how much of the violence unfolds without their ability to impose any meaningful restrictions on the incumbent.

### Qualitative results

To complement my quantitative findings, I draw on qualitative evidence from my fieldwork to demonstrate how violence is used as a tool to achieve non-contestation. The qualitative component of my research highlights why non-contestation benefits the incumbent and provides insights into the violent mechanisms behind it – nuances that quantitative data alone cannot capture. Interviews with government employees involved in local elections reveal that the non-contestation is often a product of violence orchestrated by the incumbent.

I recall accepting the nomination papers of a candidate who was bleeding from the head after being attacked by individuals concealed behind masks, while on his way here. Though injured, he was proud to file his papers. Yet, a few days later, he withdrew. Contesting is vital for parties, so avoiding elections signals something illegal is happening. – Block Development Officer^20^

The above excerpt of an interview reinforces the argument that uncontested seats are achieved through violent means. Local government officials consistently describe the use of violence to suppress opposition and prevent effective participation in elections. They assert that uncontested seats are a deliberate manipulation of the electoral process.^21^ While local election officers and civil servants are aware of the use of violence, they are often unable to intervene in such matters. Attempts to censure episodes of election violence perpetrated by incumbent-sponsored actors often leads to a demotion or transfer.^22^

Election officials often justify their inaction by citing a shortage of security arrangements. As one official explained,

We station police forces around the office (the office where nomination papers have to be filed). We cannot protect people if they are harmed on their way here. There just isn’t enough people.^23^

Even when alternative measures, such as accepting nominations via the Internet, were introduced, party workers would often discover and target those who dared to submit nominations remotely (Times of India, 2018b). This measure was eventually overturned by the judiciary after the state electoral body filed a complaint, arguing it violated electoral rules. Consequently, opposition party workers remained vulnerable to attacks when they attempted to file their papers in person (India Today, 2018).

Local rural elections are high stakes elections where control of a GP implies control of funds amounting to at least USD120,000 for a term of 5 years.^24^ My respondents spoke of multiple ways in which potential candidates can be pressured to withdraw from the election. These strategies go beyond physical harm to the candidate. Some of the strategies mentioned include the kidnapping of the candidate’s family members and vandalization of their houses.^25^ Candidates representing Leftist parties in Naskarpur GP reported that their houses were raided and vandalized (Times of India, 2018b). Fieldwork evidence from the village suggests that the attackers were armed with bombs and guns, which respondents allege were supplied by party elites.

The qualitative evidence also traces exactly how opposition actors became the targets of violence.

They [AITC party workers] set up roadblocks a few miles ahead of the BDO’s [Block Development Officer] office which prevented us from getting there and filing our nomination papers. We notified the police but no one came to help us. – Local Congress^26^ leader^27^

My cameraman and I were pelted with bricks while filming incumbent party workers violently restraining opposition candidates near the BDO’s office. As violence escalated, we sought shelter, but incumbent workers went door-to-door hunting rival party members and journalists in hiding. – Local journalist ^28^

These vignettes depict how the incumbent uses violence prior to the election to demobilize opponents and ensure that seats remain uncontested. The incumbent is keen to ensure that these violent strategies are kept under wraps to avoid sparking outrage. This point is illustrated by the attack on journalists. The incumbent in such areas has enough capacity to launch a full-scale attack upon challengers and civilians like journalists. Opposition actors often find themselves unable to counter the violence effectively or seek redress, as their complaints go unacknowledged. Additionally, opposition members are frequently falsely accused of being aggressors, leading to their arrest, which forces them to withdraw from the electoral race.^29,30^ Pre-election violence in areas under incumbent control demonstrates the party’s extensive manpower. For instance, in Bankura district, a senior BJP leader alleged that nearly 300 AITC-affiliated ‘goons’ attacked him while he attempted to file a complaint about being unable to submit nomination papers. The ability to mobilize such large numbers of party workers – capable of going door-to-door in neighboring villages to suppress opposition mobilization – is indicative of the incumbent’s dominance. Even when restrictions on assembly are imposed, incumbent-affiliated actors can operate with relative impunity.

These findings support the central claim of this article that uncontested seats are a coercive form of building partisan control. The violent removal of challengers allows the incumbent to signal its hegemony and completely reduce local opposition presence in incumbent strongholds.

## Conclusion

This article extends the conceptualization of non-contestation as a deliberate and violent tactic employed by incumbents to preserve political power. By doing so, the article presents a theory and evidence demonstrating that non-contestation can be strategically engineered by incumbents as part of their electoral strategy, rather than merely resulting from the opposition’s lack of resources or weak organization. Using the case of West Bengal, the study finds that incumbents are more likely to use violence to engineer non-contestation in areas where the incumbent is strong and has robust networks, highlighting how power is consolidated.

While the study draws on evidence from West Bengal, India, it has broader implications. Violence in local politics has been on the rise, with India overall experiencing two-thirds of violent events against local officials in 2023 in the Asia Pacific region. Such trends when coinciding with elections and emerging from countries that are not considered to be actively violent are troubling for local democracy (*ACLED*, 2024).^31^ Therefore the theory and findings of this article also have important implications for research on the emergence of subnational authoritarian regimes where incumbents routinely use violence to suppress any meaningful opposition. Future research could explore the effect of multiple election cycles and whether violence and non-contestation are useful or even required in entrenching the incumbent. Additionally, exploring other local factors, such as institutional strength, elite coalitions or the availability of violence actors, could provide a more comprehensive understanding of violence’s role in undermining democratic processes. Finally, future studies should consider how violence interacts with other undemocratic practices, such as turnout buying or opposition co-optation.

This article provides valuable insights for policymakers and election observers to identify regions vulnerable to non-contestation driven by electoral violence. By highlighting the relationship between incumbent strength and violence, it emphasizes the need for targeted measures to protect opposition candidates and voters, ensuring fairer elections. Such strategies are crucial in mitigating violence that undermines both democratic participation and the proper functioning of local governance. Reducing violence in elections not only promotes competitive elections but also ensures that ordinary citizens can benefit from accountable and effective local government.

## Notes

1. In the 2023 Philippines local elections, 7226 council president- and 1611 councillor seats were uncontested, alongside 29 incidents of election violence and 19 deaths. Similarly, Bangladesh’s 2019 and 2024 local elections saw high violence and 115 of 148 seats uncontested.

2. President’s Rule, intended for constitutional crises, has often been misused to intimidate uncooperative state governments or resolve internal party conflicts, even in the absence of genuine crises.

3. Provincial elites avoid using police or military forces due to limited control and the reputational risks of reduced plausible deniability.

4. Although voters will feel the indirect effects of violence, it acts as a clear warning to voters about the consequences of resistance and further weakens opposition support.

5. While Seeberg et al. (2020) discuss candidate violence mostly from the lens of intraparty violence, it does not mean candidate violence cannot occur between parties.

6. The Amendment obligated state governments to create a system of rural local governance.

7. Several GPs within a designated area are supervised by another elected body known as the *Panchayat Samiti* at the block level. A block is a rural administrative body earmarked for development schemes. The final tier of this system of rural governance is the supervisory body at the district level also known as the *Zilla Parishad*. Voters elect their representatives to all three tiers in a single election.

8. Some Indian states, mandate ‘party-less’ panchayat elections to prevent deepening partisan divides in communities already fractured by caste and religion.

9. The Left Front, a coalition led by the CPI(M) (Communist Party of India (Marxist)), was formed in 1977 amid the Congress’s waning popularity in West Bengal.

10. The Block Development Officer is in charge of conducting rural elections within an administrative block.

11. The article focuses on panchayat elections, as 68% of West Bengal’s population lives in rural areas, with 3,343 village councils, compared to just 108 municipalities, making these elections a higher priority for political parties.

12. Further descriptive information is reported in Online Appendix A in Table IA.

13. Codebook attached in Online Appendix C.

14. Detailed information on non-contestation via media reports is even harder to obtain. With about 3,353 local councils, each having 10–15 seats, there are approximately 40,000 seats in total. Given this scale, it is challenging to report and verify detailed non-contestation data for each individual seat.

15. This measure does not take into account seats that were won uncontested, that is, without an election.

16. Descriptive information of all control variables is provided in Online Appendix A.

17. Full table with controls is reported in the Online Appendix.

18. Centering the variables enables interpretation of main effects at average predictor values, avoiding less meaningful interpretations, such as the effect of violence when incumbent strength is zero.

19. This finding is tentative, not definitive, as provincial constituencies are larger than the local units studied here, and the elections occurred 2 years apart.

20. Interview E04270323.

21. Interview LG6270323.

22. Interview OPPO_B01321222 and Interview LG7260323.

23. Interview E03280323.

24. Local government officers claimed that GPs generate a revenue of at least USD120,000. This is not including the amounts often allocated to GPs under certain government schemes. For example, the state government announced a budget of USD36 million to local offices for the building of roads.

25. Interview with JO7121222.

26. The Indian National Congress, also known simply as the Congress is a political party in India with a widespread presence at all levels of governance.

27. Interview OPPO_CO281221.

28. Interview JO7121222.

29. Interview JO5101222 OPPO_B016151222.

30. Evidence from the 2023 panchayat elections supports these claims. For instance, a petition filed by a Bharatiya Janata Party (BJP) leader alleged that numerous BJP supporters were falsely implicated in criminal cases to bar them from participating in the election. In response, the Calcutta High Court directed the police to conduct an inquiry.

31. India, Myanmar, Mexico, Colombia and Nigeria accounted for over 40% of global violence against local officials in 2023, with subnational elections in countries like Colombia, Nigeria, India and the Philippines driving much of this violence.
